# The superficial zone of articular cartilage

**DOI:** 10.1186/s41232-022-00202-0

**Published:** 2022-05-02

**Authors:** Taku Saito

**Affiliations:** Sensory & Motor System Medicine, Graduate School of Medicine, The University of Tokyo, 7-3-1 Hongo, Bunkyo-ku, Tokyo, 113-8655 Japan

**Keywords:** Articular cartilage, Osteoarthritis, Superficial zone, Lubricin

## Abstract

The superficial zone of articular cartilage contributes to smooth joint motion through the production of proteoglycan 4 (PRG4), also known as lubricin. Recent studies indicate novel effects of PRG4 as a signaling molecule, other than a simple extracellular matrix protein. Additionally, the accumulating evidence displays that various molecules and signaling pathways are involved in regulating the superficial zone and PRG4 expression. In addition, Prg4-expressing cells include a progenitor population of articular chondrocytes. Several non-clinical and clinical studies have shown that PRG4 and related molecules are promising candidates for disease-modifying drugs for treating osteoarthritis. Since PRG4 is also expressed in the synovium, tendons, and ligaments, further studies of PRG4-related pathways and PRG4-positive cells may elucidate the mechanisms underlying joint homeostasis.

## Background

Articular cartilage is a major component of synovial joints. It serves as a low-friction, wear-resistant surface for load support, load transfer, and motion between the bones. Degeneration of articular cartilage leads to osteoarthritis (OA), the most common joint disorder. As a typical multifactorial disease, OA pathogenesis involves aging, obesity, joint instability, trauma, and genetics. Excessive mechanical loading is common among several of these factors. Therefore, several types of exercise have been recommended by a recent international guideline [1], indicating that an appropriate amount of mechanical loading may be necessary for articular homeostasis. Articular cartilage is exposed to various kinds of mechanical loading: shear stress in the superficial zone (SFZ) and compressive force in the deep zone (DZ). Accumulating evidence indicates various roles of the SFZ in maintaining the homeostasis of articular joints. In this review, we describe the differences between the SFZ and the DZ, the roles of SFZ-specific matrix protein, and the cells of the SFZ.

## The layered structure of articular cartilage

Articular cartilage consists of multiple different layers characterized by respective matrix structures. The most superficial surface is covered with the lamina splendens, a thin membrane composed of fine fibers [2]. Beneath the lamina splendens lies the tangential zone, which consists of tight bundles of collagen fibers arranged parallel to the articular surface [2]. The transitional and radial zones contain abundant proteoglycans as well as collagen [2]. The diameter of collagen fibrils becomes larger in the DZ [2]. The calcified zone constitutes the basal layer adjacent to the subchondral bone [2].

Chondrocytes in the SFZ, including the lamina splendens and the tangential zone, are flattened and thin. In the DZ, chondrocytes are cuboidal, and cell density is lower than that in the SFZ. Each cartilage layer contains characteristic matrix proteins for its respective mechanical loading pattern. The SFZ cells produce proteoglycan 4 (PRG4), also known as lubricin. Consisting of many repeated mucin-like domains, PRG4 plays a pivotal role in maintaining the lubricated surfaces of articular cartilage. Compared with the SFZ, the DZ cartilage contains thicker collagen fibrils and abundant proteoglycans including aggrecan, which contribute to resistance against compressive forces [3].

## The SFZ and PRG4

Degradation of the cartilage matrix is a crucial feature of OA pathophysiology. Cartilage degeneration is first observed as fibrillation in the SFZ [4]. Once the SFZ is disrupted, the cartilage in the DZ is subsequently degenerated [4]. Although its association with the development of general OA has not been fully revealed, PRG4 plays an essential role in maintaining articular joints. PRG4 is expressed in the lining layer of the synovium, tendons, and ligaments, as well as in the SFZ of articular cartilage [5–8]. Loss-of-function mutation of the *PRG4* gene causes camptodactyly-arthropathy-coxa vara-pericarditis (CACP) syndrome [5]. So far 37 disease-causing mutations have been identified in exons 1, 6, and 8–12 of the *PRG4* gene [9]. Patients with CACP display synovial hyperplasia and subsequent camptodactyly (flexion contractures of the phalangeal joints of fingers and toes) and arthropathy, which occur congenitally or in childhood [5]. The arthropathy in CACP patients is characterized by pain, swelling, and reduced range of motion, but it lacks inflammatory changes [5]. Rhee et al. examined *Prg4* expression during joint development [7]. *Prg4* was not expressed at E14.5 and was first detected in the SFZ at E15.5 [7]. *Prg4* was consistently expressed in the SFZ and the superficial layer of the synovium during skeletal development and after skeletal maturation [7]. Rhee et al. also created *Prg4* knockout mice and analyzed their phenotype [7]. The knockout mice displayed joint swelling and contractures [7]. Histologically, the synovium and articular cartilage were thickened, and the cartilage surface was irregular [7]. Interestingly, ectopic cartilage formation was observed in the synovium and tendons of the *Prg4* null mice [7].

## The roles of PRG4 as a signaling molecule

Besides joint surface lubrication, PRG4 works as a signaling molecule. Alquraini et al. showed an association between PRG4 and toll-like receptors (TLR) 2 and 4 [10]. The PRG4 protein binds with TLR2 and TLR4, blocking the agonist-induced activation of both TLRs [10]. In patients with OA and rheumatoid arthritis (RA), PRG4 in the synovial fluid suppresses inflammatory responses [10]. Iqbal et al. also reported an interaction between PRG4 and TLRs [11]. The PRG4 protein can bind with TLR2, TLR4, and TLR5 and decreases the activation of nuclear factor-kappa B (NF-κB) by lipopolysaccharide [11]. They also displayed that intra-articular injection of PRG4 reduced OA progression and pain-related behavior [11]. Al-Sharif et al. showed PRG4 binding to CD44 [12]. Hyaluronic acid (HA) is an essential matrix component of articular cartilage and abundantly exists in synovial fluid. As a receptor for HA, CD44 also mediates various effects of HA [13]. Both HA and PRG4 can bind with CD44, further suppressing inflammatory cytokine-induced proliferation of synovial fibroblasts in RA [12]. Alquraini et al. also reported anti-inflammatory effects of PRG4 in OA [14]. In *Prg4* knockout synoviocytes, NF-κB is activated [14]. Alternatively, exogenous PRG4 protein suppresses NF-κB [14]. Treatment with recombinant PRG4 reduced the expression of matrix metalloproteinases (MMPs) and interleukin (IL)-6 [14].

**Fig. 1:**
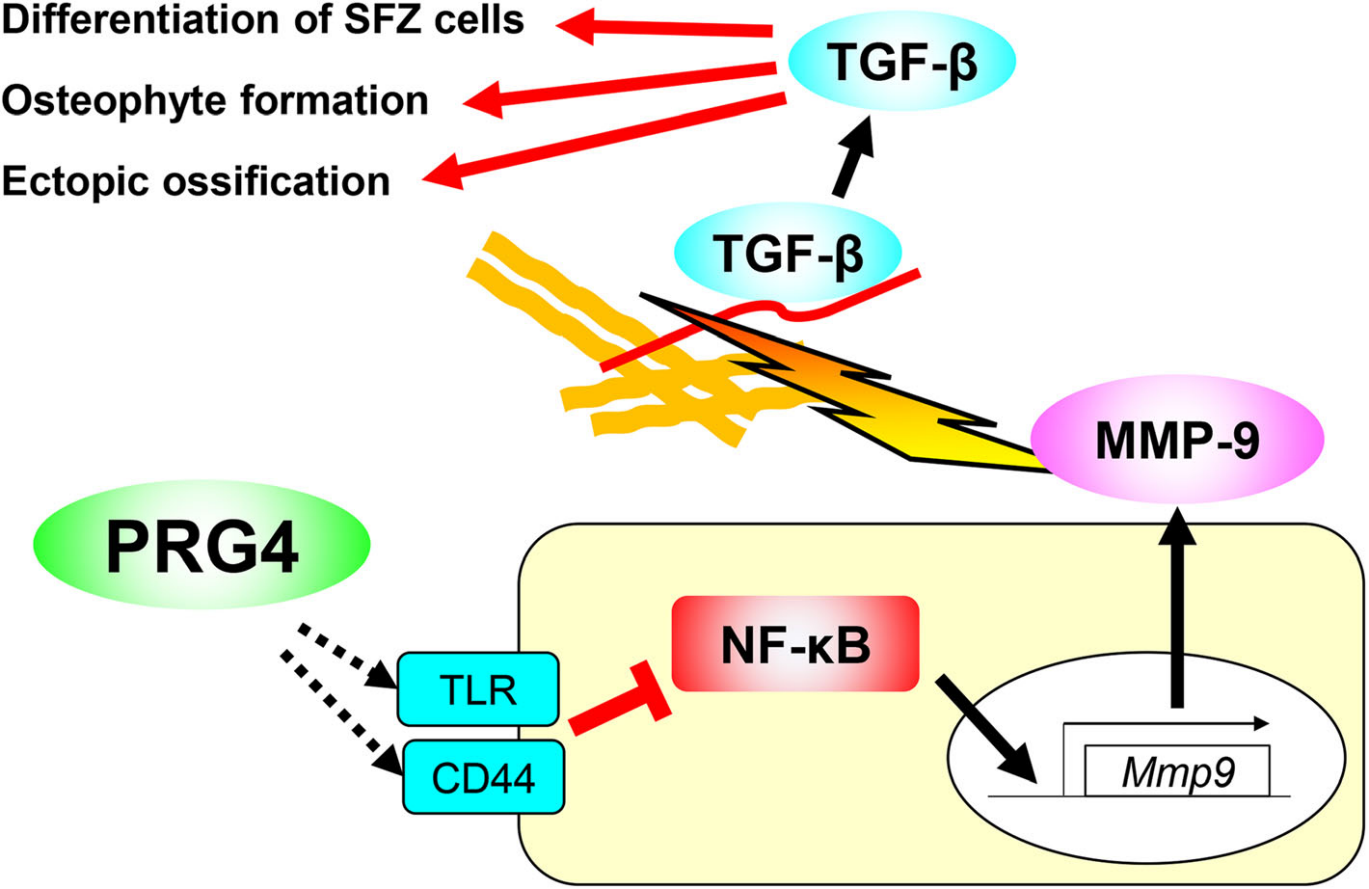
Schematic diagram representing the molecular pathway in which PRG4 suppresses the differentiation of SFZ cells, osteophyte formation, and ectopic ossification

Recently, we reported other roles of PRG4, such as modulating the differentiation of SFZ cells [15]. As described above, *Prg4* knockout mice display increased articular cartilage thickness [7]; however, the underlying molecular mechanism was unknown. We confirmed that the SFZ disappeared at eight weeks in the Prg4 knockout mice, and their cartilage was significantly thicker than that of wild-type (WT) mice [15]. In addition to the enhanced cartilage degeneration caused by the SFZ disappearance, ectopic endochondral ossification was observed with aging in the knockout mice [15]. Cell tracking indicated that *Prg4* homo-knockout SFZ cells abnormally expand to the DZ layers, compared with *Prg4* hetero-knockout cells [15]. We found that the overexpression of Prg4 transcript variant 2, which lacks enormous tandem repeats of a mucin-like sequence, intensively suppressed chondrogenic differentiation of ATDC5 cells [15]. In contrast, primary SFZ cells derived from the knockout mice showed enhanced differentiation compared with the SFZ cells of WT mice [15]. We found increased *Mmp9* in the *Prg4* knockout SFZ cells by RNA sequencing and identified that transforming growth factor-β (TGF-β) signaling was negatively regulated by PRG4 in the SFZ through suppression of NF-κB [15]. The NF-κB-MMP9-TGF-β pathway is probably responsible for the downstream action of PRG4 in suppressing the differentiation of SFZ cells, osteophyte formation, and ectopic ossification in the synovium (Fig. 1).

## Signaling pathways regulating the SFZ and PRG4

### CREB1-related pathways

In addition to the roles of PRG4, various molecules and signaling pathways have been associated with regulating the SFZ, or transcription of the PRG4 gene. Ogawa et al. identified that PRG4 expression in the SFZ of *Prg4*-reporter mice is induced by wheel running [16]. Shear stress loading by fluid flow also enhanced *Prg4* expression in vitro [16]. Prostaglandin E2 (PGE2) and parathyroid hormone-related peptide (PTHrP) are involved in the induction of *PRG4* by mechanical loading, and the cyclic adenosine monophosphate (cAMP)-protein kinase A (PKA)—cAMP response element binding protein 1 (CREB1) pathway mediates the processes [16]. Recently, we identified transient receptor potential vanilloid channel 2 (TRPV2) as one of the mechano-sensors involved in PRG4 induction [17]. TRPVs are Ca^2+^-permeable channels, and some members mediate intracellular Ca^2+^ current via mechanical stimuli. TPRV2 is expressed in the superficial and middle layers of normal articular cartilage in humans and mice, but its expression decreases with aging or OA progression [17]. Chondrocyte-specific knockout of *Trpv2* results in accelerated OA in surgically-induced and aging models, accompanied by decreased expression of *Prg4* [17]. Mechanical stimulation-induced changes in intracellular Ca^2+^ influx are markedly impaired in *Trpv2* knockout chondrocytes [17]. In vitro experiments using inhibitors indicate that calmodulin-dependent protein kinase kinase (CaMKK) and probably CaMKIV mediate *PRG4* induction by CREB1 downstream of the TRPV2 pathway [17].

### WNT signaling pathway

WNT signaling performs various essential roles in the development and homeostasis of cartilage and bone. Koyama et al. showed Wnt/β-catenin signaling is activated in developing joints during skeletal growth [18]. β-catenin knockout by *Col2a1-Cre* or *Gdf5-Cre* leads to a marked decrease of *Prg4* in the SFZ of mouse embryos [18]. Morphology and proliferation are impaired in β-catenin-knockout SFZ cells, and mRNA levels of *Prg4* depend on Wnt/β-catenin signaling activity [19]. We previously showed that Wnt/β-catenin signaling is activated specifically in the SFZ of articular cartilage in adult mice [20]. Tamoxifen-induced β-catenin knockout in *Prg4* positive SFZ cells of adult mice leads to enhanced OA accompanied by an impaired SFZ, while β-catenin stabilization by exon 3 deletion inhibited OA development [20]. Among Wnt ligands, *Wnt5a*, *Wnt5b*, *Wnt9a*, and *Wnt16* are highly expressed in the SFZ cells [20]. mRNA levels of *Wnt5a*, *Wnt5b*, and *Wnt9a* are increased by shear stress loading, and recombinant WNT5A and WNT5B increased *Prg4* expression [20]. Both Creb1 expression and its phosphorylation are enhanced by shear stress loading or stimulation of Wnt/β-catenin signaling [20]. Nalesso et al. reported roles for WNT16 in OA [21]. Wnt16 expression in the SFZ is enhanced during OA development, and *Wnt16* knockout mice displayed severer cartilage degeneration in a surgical OA model [21]. Prg4 expression was decreased by *Wnt16* knockout, while *Prg4* was increased by recombinant WNT16 treatment [21].

### EGFR and other signaling pathways

Epidermal growth factor receptor (EGFR) signaling also plays several roles in the SFZ. EGFR is dominantly expressed and phosphorylated in the SFZ of normal articular cartilage in mice and humans, while they are inhibited in OA cartilage [22]. Disruption of EGFR signaling enhances OA [22]. EGFR signaling is required for maintaining the SFZ cells, and *Prg4* expression is induced by TGF-α, one of EGFR’s ligands [22]. Reddi et al. reported that TGF-β1 and bone morphogenetic protein-7 (BMP-7) are potent inducers of PRG4 in mesenchymal stem cells, SFZ cells, and synovial explants [23–25]. Cell division cycle 42 (CDC42), a small GTPase of the Rho-subfamily, is also required for PRG4 expression [26]. Actin and myocardin-related transcription factor-A are involved in PRG4 regulation by CDC42 [26]. Yes-associated protein and transcriptional co-activator with PDZ-binding motif are associated with PRG4 induction downstream of CDC42 [27].

### Transcription factors

In addition to these signaling pathways, several transcription factors have been identified as regulators of the SFZ cells or PRG4 expression. As described above, Creb1 is a potent transcription factor of the *PRG4* gene. Recently, Creb5 was identified as a novel transcription factor upstream of *PRG4* [28]. Creb5 is specifically expressed in SFZ cells, and it is required for TGF-β and EGFR signaling to induce *Prg4* expression [28]. Creb5 directly binds to two proximal enhancers of the *Prg4* gene [28]. Forkhead box O (FoxO) proteins are transcription factors associated with the maintenance of stem or progenitor cell populations. Matsuzaki et al. showed that chondrocyte-specific knockout of *FoxO1*, *FoxO3*, and *FoxO4* resulted in thickened articular cartilage at 2 months but caused early-onset OA at 4 months [29]. In the triple FoxO knockout mice, the number of SFZ cells decreased at 1 month, and the proliferation of chondrocytes was enhanced [29]. Single knockout of *FoxO1* displayed a similar but milder phenotype than the triple FoxO knockout mice [29]. *Prg4* expression was markedly decreased in the triple FoxO knockout chondrocytes, and the overexpression of FoxO1 induced *Prg4* synergistically with TGF-β [29]. High-mobility group box protein 2 (HMGB2) is expressed in the SFZ of adult mice, and its expression decreases with aging [30]. *Hmgb2* knockout mice exhibit enhanced OA [30]. *Prg4* expression is maintained in 2-month-old *Hmgb2* knockout mice, but it is reduced at 6 months [30]. HMGB2 maintains the SFZ through supporting cell survival; however, it is not likely that HMGB2 directly regulates *PRG4* transcription [30]. Runt-related transcription factor (Runx) family members are expressed in chondrocytes and regulate skeletal formation. Among Runx1-3, Runx1 is expressed in the superficial and middle zones of mouse articular cartilage, and chondrocyte-specific knockout enhances OA progression [31]. Although the interaction of Runx1 and Prg4 is unknown, Runx1 contributes to the homeostasis of articular cartilage via suppression of chondrocyte maturation or hypertrophic differentiation [31].

## Progenitors in SFZ

Kozhemyakina et al. showed the properties of Prg4-expressing cells as articular cartilage progenitors [8]. They mated *Prg4-Cre*^*ERT2*^ and *Rosa26-lacZ* mice and performed cell tracking experiments. When tamoxifen was injected at E17.5, LacZ-positive cells were observed in the SFZ at 1 month and expanded to the DZ layers with aging [8]. When tamoxifen was injected at 1 month, LacZ-positive cells expanded only to the middle layer, above the tide mark [8]. They concluded that *Prg4*-expressing cells in the developing joint at E17.5 give rise to chondrocytes in all regions of articular cartilage [8]. Meanwhile, the properties of *Prg4*-expressing progenitors seem to change with aging.

## Development of novel OA therapies

Some of the superficial layer-related molecules may be useful for OA therapy, of which PRG4 itself is a potent candidate. Jay et al. reported that intra-articular injection of PRG4 derived from human synoviocytes in culture suppressed cartilage degradation and pain-related behavior in a rat model of the anterior cruciate ligament (ACL) [32]. BMP-7 is a cartilage-protective cytokine and a PRG4 inducer, as mentioned above. Intra-articular injections of BMP-7 inhibit OA progression in rabbits with ACL resection [33], and a phase 1 study of BMP-7 on OA patients has reported acceptable safety and tolerability [34]. Fibroblast growth factor (FGF)-18 is also a chondroprotective cytokine, which is expressed in the SFZ [35] and upregulates *PRG4* expression in calf chondrocytes [36]. Intra-articular injection of recombinant FGF-18 suppressed OA development in a rat model [35]. Recently, the long-term results of a phase 2 study displayed that recombinant human FGF-18 (Sprifermin) modifies structural progression in knee OA [37, 38].

## Conclusions

**Fig. 2:**
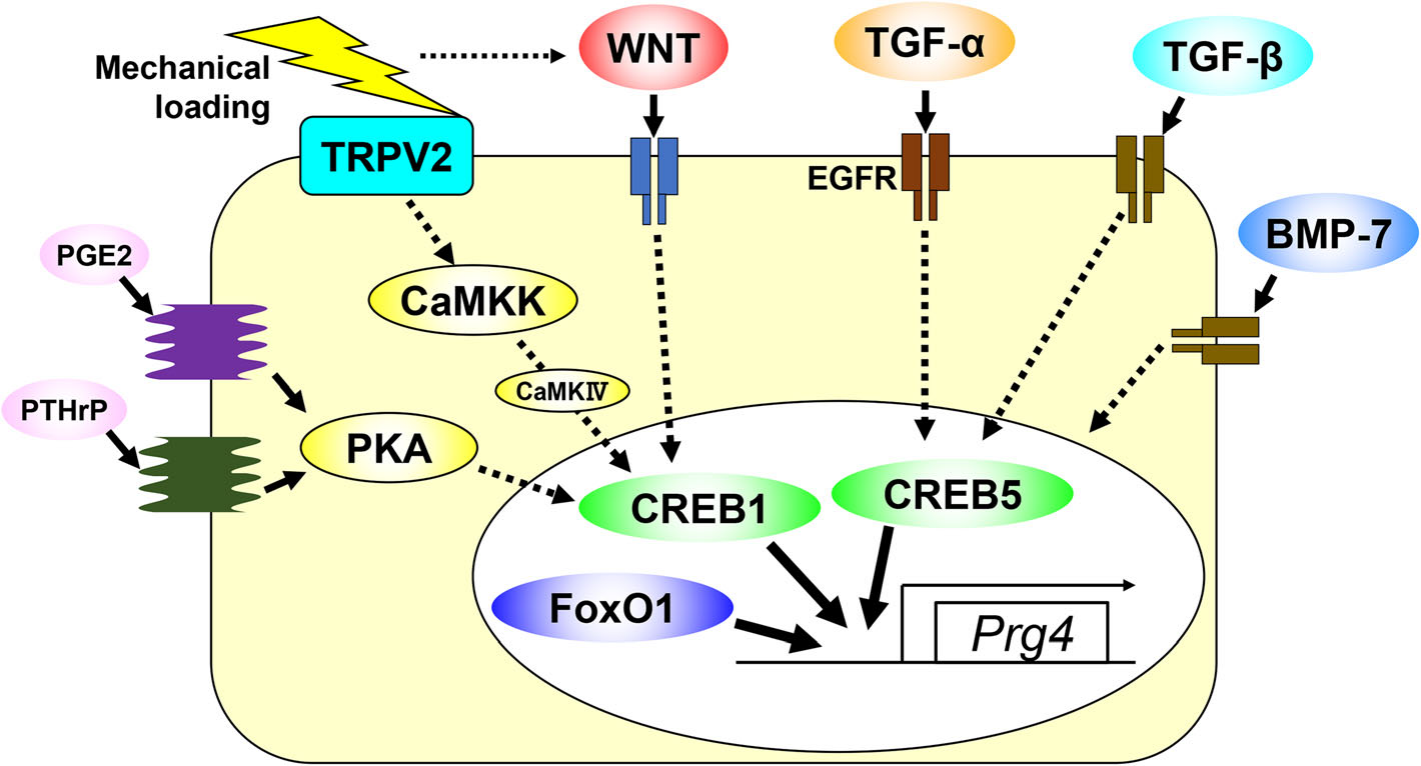
Schematic diagram representing the molecular pathways that regulate PRG4 expression

The accumulating evidence shows the essential roles of SFZ cells and PRG4 in articular cartilage homeostasis and the various molecules and signaling pathways that regulate them (Fig. 2). Some of these findings may lead to novel therapeutic methods for OA. The latest technologies, such as single-cell RNA sequencing, could further reveal the mechanisms underlying joint homeostasis. Considering that PRG4 is expressed in the synovium, tendons, and ligaments, studies of the SFZ and PRG4 may be widely useful for understanding the locomotive organ system.

## Data Availability

Not applicable

## Abbreviations

OA: Osteoarthritis
SFZ: Superficial zone
DZ: Deeper zone
PRG4: Proteoglycan 4
CACP syndrome: Camptodactyly-arthropathy-coxa vara-pericarditis syndrome
TLR: Toll-like receptors
RA: Rheumatoid arthritis
NF-κB: Nuclear factor-kappa B
HA: Hyaluronic acid
MMP: Matrix metalloproteinase
IL: Interleukin
WT: Wild type
TGF: Transforming growth factor
cAMP: Cyclic adenosine monophosphate
CREB: cAMP response element binding protein
TRPV2: Transient receptor potential vanilloid channel 2
EGFR: Epidermal growth factor receptor
BMP-7: Bone morphogenetic protein-7
CDC42: Cell division cycle 42
FoxO: Forkhead box O
HMGB2: High-mobility group box protein 2
Runx: Runt-related transcription factor
ACL: Anterior cruciate ligament
FGF: Fibroblast growth factor
